# Signatures of Demography and Recombination at Coding Genes in Naturally-Distributed Populations of *Arabidopsis Lyrata* Subsp. *Petraea*

**DOI:** 10.1371/journal.pone.0058916

**Published:** 2013-03-12

**Authors:** Cynthia C. Vigueira, Brad Rauh, Thomas Mitchell-Olds, Amy L. Lawton-Rauh

**Affiliations:** 1 Department of Genetics and Biochemistry, Clemson University, Clemson, South Carolina, United States of America; 2 Department of Biology, Washington University, St. Louis, Missouri, United States of America; 3 Departments of Genetics and Biochemistry, and Horticulture, Clemson University, Clemson, South Carolina, United States of America; 4 Department of Biology, Duke University, Durham, North Carolina, United States of America; University of Umeå, Sweden

## Abstract

Demography impacts the observed standing level of genetic diversity present in populations. Distinguishing the relative impacts of demography from selection requires a baseline of expressed gene variation in naturally occurring populations. Six nuclear genes were sequenced to estimate the patterns and levels of genetic diversity in natural *Arabidopsis lyrata* subsp. *petraea* populations that differ in demographic histories since the Pleistocene. As expected, northern European populations have genetic signatures of a strong population bottleneck likely due to glaciation during the Pleistocene. Levels of diversity in the northern populations are about half of that in central European populations. Bayesian estimates of historical population size changes indicate that central European populations also have signatures of population size change since the last glacial maxima, suggesting that these populations are not as stable as previously thought. Time since divergence amongst northern European populations is higher than amongst central European populations, suggesting that the northern European populations were established before the Pleistocene and survived glaciation in small separated refugia. Estimates of demography based on expressed genes are complementary to estimates based on microsatellites and transposable elements, elucidating temporal shifts in population dynamics and confirming the importance of marker selection for tests of demography.

## Introduction

Demographic factors shape baseline genetic variability within and between populations and can obscure detection of molecular evolution signatures and identification of adaptive alleles [1], [2]. Demographic factors include population structure, effective population size changes and gene flow between populations, all of which can dramatically impact the levels of genetic diversity across the entire genome [1], [3]. Selection acts on genome regions that carry adaptive alleles [2]. The use of multiple unlinked loci is now commonly employed in population genetic studies to capture the levels of genetic diversity which have been shaped by the demographic history of that population [4]–[9]. Although this approach has been widely accepted, the extent to which demography can shape genetic variability at coding genes across the genome has not been well tested in naturally occurring populations, but see [6], [8], [10]. This is most likely due to the lack of natural populations in which demography can be separated from other processes, such as selection and adaptation as well as lack of extensive sampling focused on attributes specific to alternative demographic histories rather than tests for evidence of adaptive evolution in candidate genes.

Understanding the role of demography in genetic variation is important for the implementation of population genetic theory to tease apart selection and population structure and size fluctuations. Inferring the best fit model explaining the source and patterning of genetic variation in a system is hinged on an understanding of the demographic history which shapes the baseline variation of the genome. However, baseline genome variation falls in a distribution of values. Demography impacts the distribution of baseline variation across the genome, shifting all loci including outliers [11], [12]. Therefore, it is necessary to determine the distribution of genetic diversity at random loci across the genome to represent the baseline variation, specifically coding genes because they share the same mutation constraints, before selection on candidate genes can be tested.

Dissecting the genetic impacts of demography is further confounded by the temporal fluctuations of population size and population connectivity. Within several generations, the genome-wide impacts of bottlenecks may no longer be statistically detectable (loss of rare alleles, increases in inbreeding coefficients, etc.) [3]. Recent population size change is therefore difficult to fully assess from stochastic accumulation of mutations due to drift and recombination. The initial standing levels and patterns of genetic variation amongst and within populations also shape the impact of population size changes [3]. This makes it difficult to measure impacts of demography without a ‘control’ population that has not experienced the same demographic change. Finally, the types of markers used are very important for understanding the impact of demography. The mutation rate, substitution model, and occurrence of recombination within and surrounding a marker directly impacts how quickly alleles originate and can freely move around within and between populations [13]–[15]. It is for these reasons that few studies have focused specifically on empirical tests of the genome-wide impacts of alternative demographic histories, but see [5], .

Determining the extent that demography can impact genetic variation in naturally-distributed populations requires closely related populations with differing demographic histories as well as an excellent evolutionary and ecological background on both the population and the species levels. *Arabidopsis thaliana* (*A. thaliana*) has long been the model plant used in genetic and developmental studies due to its small genome size, ease and quickness of growth and propagation in laboratory settings, and plasticity to genetic modifications. This model system status has resulted in a large knowledge base of gene function and a sequenced, well characterized genome. Genus *Arabidopsis* is also a model for speciation and evolutionary ecology due to the interesting life history differences present in populations of closely related species [21]–[25].

The closest relative of *A. thaliana* is *A. lyrata subsp. petraea (A. l. petraea)* [26], an out-crossing perennial with restricted habitat preference for low competition, dolomitic rocky outcrops. Due to the species’ specific habitat preference, populations have not been greatly disturbed by human or animal migrations. Populations of *A. l. petraea* have therefore been found to be stable with isolation by distance detected in microsatellite loci [5], chloroplast and mitochondrial loci [27] and nuclear loci [17], [28]. Self-incompatibility in this species results in higher levels of heterozygosity than its inbreeding cousin, *A. thaliana* [24].

European populations of *A. l. petraea* have differing demographic histories [5], [17]. Northern populations are located in regions that were glaciated during the Last Glacial Maximum (LGM) [28]. This history has most likely resulted in population bottlenecks followed by post-glacial expansion. The central European populations, on the other hand, were not located in glaciated regions and thus are presumed to have remained stable in population size and distribution throughout the Pleistocene. A study using allozyme markers [28] cites evidence of strong demographic impacts on *A. l. petraea* across the entire range. This finding is in contrast to the results cited using microsatellite markers, which indicate stability in central European populations [5]. The natural history, an array of available genetic tools, and the absence of other confounding factors (such as human disturbance) makes European populations of *A. l. petraea* a great system to study the impacts of demography on genetic diversity of naturally-distributed populations.

We developed and utilized expressed haplotypes of single copy nuclear loci (SCNL) to determine the signatures of demographic histories between populations of *A. l. petraea*. Coding regions of the genome are better suited to divergence population genetics than are microsatellites, transposable elements or chloroplast markers, due to the availability of more consistent and predictive mutation accumulation models, especially for coalescent-based analyses. We estimated the baseline impact of alternative demographic histories on coding gene diversity as an independent distribution for use by the ecology and evolutionary genetics community to dissect the impact of selection in relevant functionally-important candidate genes in genus *Arabidopsis*. This analysis can also serve as a reference point for future comparisons across different systems to test broader comparative genomics hypotheses.

## Materials and Methods

### Population Sampling and DNA Sequencing

**Figure 1:**
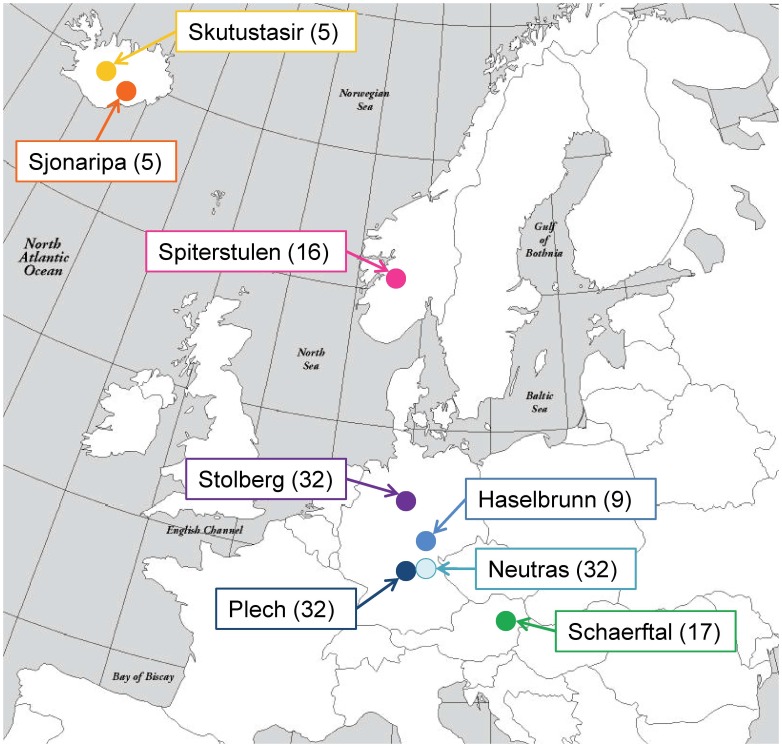
Map of *Arabidopsis lyrata* subsp. *petraea* sampling in Europe. The number of sampled individuals from each population is in parentheses. Exact locations for each of the samples are as follows: Skutustasir (65°34′N, 17°08′W), Sjonaripa (64°02′N, 16°56′W), Spiterstulen (61°38′N, 8°24′E), Stolberg (51°30 N, 10°56′E), Plech (49°38′N, 11°31′E), Neutras (49°32′N, 11°33′E), Haselbrunn (49°47′N, 11°25′E), and Schaerftal (47°55′N, 15°59′E).

No specific permits were required for the described field studies, no localities were in protected areas, and no species in this study are endangered or protected. Seeds were collected from 8 populations of *A*. *l*. *petraea* in northern and central Europe for a total of 148 sampled plants (Figure 1). Seeds were germinated on media and transferred to soil containing peat moss. They were then grown in a growth chamber with 12 hours of light per day. Once mature, leaf tissue was collected for DNA extraction. Genomic DNA was extracted using Macherey-Nagel NucleoSpin 96 Plant DNA extraction kits (Duren, Germany). Twenty-one ecotypes of *A. thaliana*, selected to represent the range of diversity in *A. thaliana* by representing each clade present on a SNP-based phylogeny [29], were included as an outgroup for analysis.

Single copy nuclear loci (SCNL) were developed as markers using searches of the *A. thaliana* published genome (http://www.arabidopsis.org) to do the following: sort genes by length of longest exon, remove genes with sequences consistent with transposable element histories, include only genes that are expressed (have ESTs), and remove genes with too much nucleotide sequence similarity precluding distinction between gene copies versus alleles for PCR amplification simplicity. Only regions with long exons (greater than 500 basepairs) were considered. Genes that contained transposable elements were removed from the list to avoid confounded mutation models and evolutionary histories in coalescent models. Next, genes without representatives in the EST library were removed. Finally, to verify single copy status, only genes that had little similarity to the closest BLAST hit were retained using the reference point similarity score of paralog and ortholog detectability in gene families in other systems with which we have published [30]. This screen produced 23 loci, six of which were randomly selected for this study and represent separate chromosomal locations (Table S1). Primers were designed to amplify 400–800 bp of exon for each gene. All genes were amplified by Polymerase Chain Reaction (PCR) using standard conditions with GoTaq polymerase. Magnesium chloride (MgCl_2_) concentrations and annealing temperatures were adjusted as necessary amplification. Single bands were verified by gel electrophoresis. Excess primers and dNTPs were removed by Exonuclease and Antarctic Phosphatase treatment. Direct sequencing reactions were carried out with both forward and reverse primers on PCR products using ABI Big Dye protocols by the Max Plank Institute for Chemical Ecology and the Clemson University Genomics Institute (CUGI).

### Data Processing

Sequences were aligned and checked for quality using CodonCode Phred/Phrap and BioLign version 4.0.6.2 (Tom Hall, http://en.bio-soft.net/dna/BioLign.html). Alignments were trimmed to contain only high quality base calls (quality score of 30 or better) and to insure the same length for all sequences. Heterozygous sites were called by eye using the presence of a double peak from sequence reads in both directions. Sequences were doubled for each individual and pseudo-haplotypes were created by random assignment of heterozygous base calls. PHASE version 2.1 [31], [32] was used to assign haplotypes to the genotypic data set. The program was run five times for each dataset (both on whole data sets and individual population data sets) with random number seeds. Whole data set runs resulted in lower numbers of ambiguous sites and higher overall haplotype probability scores. Haplotypes with consistently high probabilities were accepted for further analysis. The output was checked by cloning two randomly chosen individuals and sequencing eight clones for each locus. It was also checked with trio data from a subset of individuals in the Plech and Spiterstulen populations. The trio data consisted of parental sequences (two individuals from the population that were crossed in the lab) and sequences from four to six offspring from each cross. These data were used to determine the true haplotypes of the parental plants by determining which SNP calls are inherited together in the offspring. PHASE called all checked haplotypes correctly. Lists of ambiguous sites for haplotypes with less than a 0.75 probability were compiled. Less than 1% of sites fell into this category. GenBank accessions for all sequences used in this study are JX858007-JX859883.

### Data Analyses

Haplotype networks for each locus were estimated using statistical parsimony in TCS version 1.21 [33]. Connection limits were fixed up to 60 steps in order to connect *A. thaliana* ecotypes to the networks. Networks were saved in TCS format and manually converted to graphs in Microsoft Powerpoint.

DnaSP version 4.0 [34], [35] was used to calculate standard indices including numbers of segregating sites, haplotype diversity, nucleotide diversity at silent sites (π) using the Juke’s Cantor correction [36] and Watterson’s θ at silent sites [37]. Neutral-equilibrium models and signatures of population size change were tested by estimating Tajima’s *D* [38], Fu’s F*_S_* [39] and Rozas’ R*_2_* [40] statistics in DnaSP using 10,000 coalescent simulations to assess significance. Bonferroni corrections for multiple tests were used to determine appropriate *P*-value cutoffs for statistical significance.

The best fit mutational models were identified for each gene using FINDMODEL (available at http://www.hiv.lanl.gov/content/sequence/findmodel/findmodel.html) using Akaike Information Criteria (AIC) as in MODELTEST [41]. The best fit model for *Phr2* was TrN [42]. GTR [43] was the best fit for all other genes. The more complex GTR model is not available in most analysis software, so we used the model hierarchy from MODELTEST to choose the simpler TrN model for analyses in Arlequin version 3.1 (Gamma distribution shape parameters from the original model were used in ARLEQUIN, [44]) and the HKY [45] model for IMa analysis.

Arlequin version 3.1 [44] was utilized to estimate Ф_ST_ among populations using analysis of molecular variance (AMOVA), pairwise Ф_ST_ between each population, exact tests for population differentiation, and Hardy-Weinberg equilibrium. We used 10,000 permutations to assess significance of AMOVAs and pairwise Ф_ST_, while 100,000 steps were used to assess significance of exact tests and HWE. Bonferroni corrections for multiple tests were used to determine appropriate *P*-value cutoffs for statistical significance.

Recombination break points in each gene were determined using the 4 gamete test [46]. Estimation of the population recombination parameter was calculated as Hudson’s 4 N*_C_* [47]. Both recombination tests were performed using SITEs [48].

Best fit population structure was inferred using STRUCTURE version 2.2 [49]. Each unique haplotype was assigned a number, with two numbers representing the genotype of the individual at each locus. Five permutations for each number of populations (K) were set from 1 to 10 with 100,000 repetitions and a burn-in period of 10,000 steps. Log likelihoods for each run were compared to determine the best fit number of populations. Delta K [50] was also calculated using StructureHarvester [51]. The same K value was determined to be the best fit in both cases. Distruct [52] was used to produce graphical images of the STRUCTURE output.

BEAST version 1.4.8 [53] was used to construct Bayesian skyline plots (BSPs) for effective populations sizes (N*_ef_*) over time. Each run used the HKY mutation model and a strict molecular clock with a 1.5×10^−8^ substitutions/site/generation. The mutation rate is based on that estimated for the *Brassicaceae* family [54]. The chain was run for 100,000,000 steps and increased in cases of low Effective Sample Size (ESS) values or poor distributions of posterior probabilities. These were determined using Tracer version 1.4, which was also used to reconstruct the BSPs. Due to low diversity and small sample sizes in the Icelandic populations, BEAST could not determine effective population sizes into the past. Therefore, these two populations were not included in this analysis.

IMa [55], [56] was used to simultaneously estimate effective population sizes (N*_ef_*), migration between populations (N*_ef_m*), ancestral population size (N*_ef_*A) and time since divergence (T). All populations were analyzed on a pairwise basis. Longest non-recombining blocks (LNRB) were determined from the 4 gamete test in SITEs, and used for all analyses in IMa. Each population comparison was run in M-mode with wide value cutoffs for all parameters. After the initial run, three runs with different random number seeds and smaller cutoff values that were based on the distribution of parameter values from the first run. All runs had 50,000,000 MCMC steps after a burn-in of 100,000 steps. Each run had 10 chains with a mixing rate of 5 chain swaps per step. All three M-mode outputs are checked for convergence and nested models were tested in L-mode. Using nested models, which tests for likelihood of data fitting the model with and without migration, we found that in all cases the models that included migration fit the data better than the no migration models. Values were scaled based on a substitution rate of 1.5×10^−8^ [54] and a generation time of one year [24], although a longer generation time could be possible. A slower mutation rate of 7×10^−9^ has been calculated in *A. thaliana* [57]. Demographic parameters were also calculated with both mutation rates and there was less than an order of magnitude difference between the estimates. We report the calculations from the 1.5×10^−8^ mutation rate. All IMa runs were computed on the Condor cluster at Clemson University using primarily an extensive web-enabled system to simultaneously manage and monitor performances of each set of input priors.

## Results

### Neutral-equilibrium Model Parameters and Recombination

After Bonferroni corrections, no estimates of Tajima’s *D*, Fu’s F*_S_* or Rozas R*_2_* were significantly different from neutral-equilibrium model expectations (Table 1). Only two exceptions from Hardy-Weinberg Equilibrium were found in the data set. Those are in the Spiterstulen population at *Cori1* and in the Haselbrunn population at *Phr2*. In both cases, the deviation was due to lower than expected levels of heterozygosity at each locus within these populations.

**Table 1:** Tests for locus-specific deviations from neutral-equilibrium and demographic effects by population.

Population	Gene	Tajima's *D*	Fu's F*_S_*	R*_2_*	Obs.Het	Exp.Het
Skutustasir	Chs	0.93158	0.29979	0.2278	0.2	0.68889
	Cip7	0.02107	0.5901	0.1778	0.8	0.62222
	Cori1	0.85057	0.62543	0.2667	0	0.53333
	Fdh	1.75514	0.28115	0.263	0.4	0.84444
	Fnr	1.29017	1.20973	0.241	0.66667	0.93333
	Phr2	2.1055	4.64564	0.2617	0.2	0.68889
Sjonaripa	Chs	−0.1774	−2.53954	0.1804	0.75	0.78571
	Cip7	1.30268	1.02917	0.2667	0	0.5333
	Cori1	0.50437	1.72632	0.2143	0	0.71429
	Fdh	N/A	N/A	N/A	N/A	N/A
	Fnr	−0.83077	−1.1853	0.1329	0.4	0.84444
	Phr2	1.4488	2.08349	0.2679	0.75	0.53571
Spiterstulen	Chs	1.20043	1.44255	0.2218	0.375	0.52419
	Cip7	−0.41804	3.61447	0.108	0.875	0.6996
	Cori1	−0.18233	1.36215	0.1146	0.1875*	0.55847
	Fdh	0.69611	2.77332	0.1467	0.9375	0.70363
	Fnr	−0.92599	8.72007	0.0914	0.5	0.44556
	Phr2	−0.32894	−0.25677	0.1239	0.4375	0.36492
Stolberg	Chs	0.74139	−0.1996	0.1523	0.46875	0.5377
	Cip7	−0.6089	1.68321	0.0969	0.625	0.70188
	Cori1	−0.41439	−0.3915	0.0797	0.21875	0.30407
	Fdh	1.86759	−0.06892	0.1702	0.84375	0.87996
	Fnr	0.28758	2.863	0.1137	0.875	0.86161
	Phr2	0.20354	1.52196	0.1135	0.53125	0.55308
Plech	Chs	2.05689	2.55783	0.178	0.6875	0.80952
	Cip7	1.06535	−0.3815	0.1417	0.71875	0.83532
	Cori1	0.69904	−6.67003	0.133	0.8125	0.90526
	Fdh	1.90093	−1.39821	0.17	0.875	0.92907
	Fnr	1.30985	6.43954	0.1419	0.5	0.72123
	Phr2	1.32699	−2.68822	0.1485	0.84375	0.9494
Haselbrunn	Chs	2.39129	6.3879	0.2386	0.44444	0.60784
	Cip7	−0.04113	−0.87789	0.1409	0.77778	0.90196
	Cori1	−0.36976	−3.53241	0.1262	0.77778	0.86928
	Fdh	0.83232	−0.55272	0.1718	1	0.9281
	Fnr	0.65101	6.83191	0.1737	0.6667	0.75163
	Phr2	1.90114	0.93035	0.2327	0.66667*	0.87582
Neutras	Chs	2.29309	3.67186	0.1863	0.84375	0.85813
	Cip7	0.23291	1.06614	0.114	0.78125	0.74504
	Cori1	−0.48172	−2.40496	0.0879	0.65625	0.70238
	Fdh	1.45202	6.5347	0.1542	0.8125	0.80804
	Fnr	0.12969	9.38409	0.1104	0.21875	0.22569
	Phr2	1.78714	2.27902	0.1723	0.78125	0.75645
Schaerftal	Chs	2.39786	7.78935	0.2115	0.70588	0.74866
	Cip7	0.44989	−0.27644	0.1372	0.64706	0.69162
	Cori1	−0.48172	−4.05794	0.0984	0.64706	0.67558
	Fdh	0.68951	0.75179	0.1439	0.70588	0.88235
	Fnr	2.55967	5.35621	0.2095	0.82353	0.87344
	Phr2	−0.23219	0.27078	0.1105	0.47059	0.67023

* Significant after Bonferroni corrections.

Tests completed in DnaSP and Arlequin.

**Figure 2:**
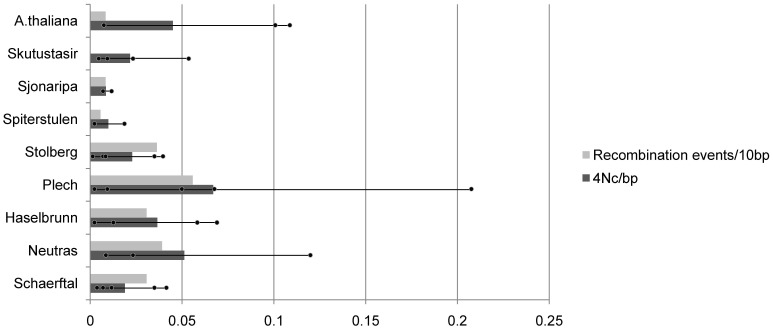
Recombination rate by population. Recombination events/10 bp is the number of recombination events counted for each gene (from the 4 gamete test) divided by the length of the gene in basepairs and multiplied by 10. These are averaged across all 6 loci. Average 4 N*_C_*/bp across 6 loci is shown by the bar. The dots indicate estimates for each locus.

The northern populations had reduced levels of recombination events per basepair than did the central populations (Figure 2). DNA sequences for all six loci in the Plech population had the highest rate of recombination, with the most detected recombination events and the highest average population recombination parameter. These values were impacted mostly by one highly recombining locus, *Fdh*.

### Population Diversity

**Figure 3:**
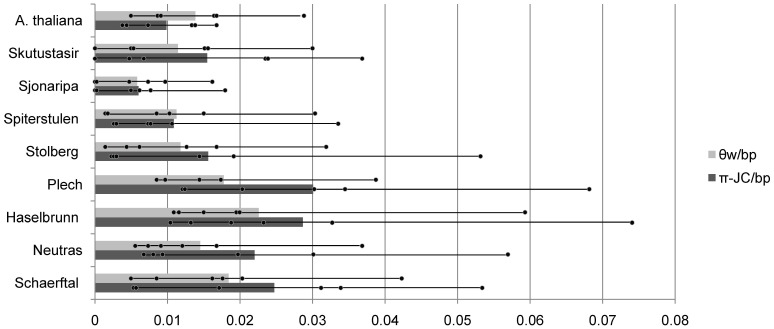
Nucleotide sequence diversity statistics by population. Each estimate of Watterson’s theta per basepair (θw/bp) and nucleotide diversity with Juke’s Cantor correction per basepair (π-JC/bp) calculated at silent sites. Average estimates across 6 loci are indicated as bars and dots indicate estimates for each locus.

Consistent with a more recent population bottleneck, average silent site nucleotide diversity (π) and Watterson’s θ are much lower in northern European populations compared to central European populations (Figure 3). The Sjonaripa population has the lowest estimates of sequence diversity amongst loci, while the Plech population has the highest. Average haplotype diversity is also lower in the northern populations (results not shown).

**Figure 4:**
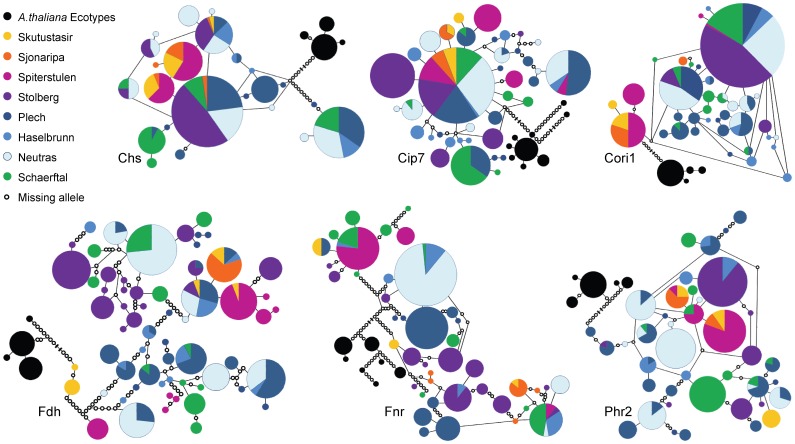
Haplotype networks for each locus. Each circle represents a single haplotype with divisions based on the proportion contributed by each population. Open circles represent inferred missing haplotypes. The connections of *A. thaliana* haplotypes (in black) into the networks are shortened to fit (greater than 30 steps for all networks). Networks constructed in TCS.

Haplotype networks (Figure 4) indicate differing patterns of diversity for each locus. Ancestral alleles for *A. l. petraea* can be inferred by the closest connections to the *A. thaliana* ecotypes. These connections are most often to central populations, but the *Cori1* and *Fdh* networks have these connections with northern populations. This could be reflective of older divergence times between northern and central populations. The most common haplotype (largest circle) for each gene is made up of a high percentage of central European individuals.

### Population Structure

Analyses of molecular covariance (AMOVAs) revealed highly significant Ф_ST_ values for all 6 loci (Table 2) with higher within population variation than between population variation in all cases. Mean and median values of Ф_ST_ are slightly different indicating that none of the 6 loci are greatly skewing these values. Average pairwise Ф_ST_ estimates were higher among the northern populations than among the central populations (Table 3). However, both Ф_ST_ estimates and exact tests for population differentiation had fewer significant values between the northern populations than amongst the central populations, indicating that the smaller sample sizes (especially in the Icelandic populations) are insufficient to detect significant differences using this test.

**Table 2:** Analysis of Molecular Variance (AMOVA) results for population structure by locus.

Locus	Source of Variation	d.f.	S.S.	Variance components	% variance	Ф_ST_
Chs	among pops	7	149.13	0.55430 Va	21.26	0.21264**
	within	286	587.01	2.05248 Vb	78.74	
Cip7	among pops	7	86.26	0.31000 Va	17.54	0.17543**
	within	288	419.63	1.45706 Vb	82.46	
Cori1	among pops	7	122.94	0.49626 Va	43.54	0.43536**
	within	282	181.50	0.64362 Vb	56.46	
Fdh	among pops	7	245.29	0.90761 Va	21.2	0.21204**
	within	287	967.96	3.37268 Vb	78.8	
Fnr	among pops	7	583.78	2.31444 Va	38.13	0.38133**
	within	284	1066.42	3.75498 Vb	61.87	
Phr2	among pops	7	161.93	0.61357 Va	25.19	0.2519**
	within	286	521.14	1.82217 Vb	74.81	
					**Mean**	**0.27812**
					**Median**	**0.23227**

** *P*-value<0.001.

Degrees of freedom (d.f.), sum of squares (S.S.), percent of total variance (% variance), and population differentiation (ФST) were estimated in Arlequin. All loci have significant among population variation. Mean and median ФST values are in bold.

**Table 3:** Pairwise inferred population histories from IMa and Arlequin.

Population 1	Population 2	T (years)*	N*_ef_* A*	N*_ef_m*1* Pop2→Pop1	N*_ef_m*2* Pop1→Pop2	Average Ф_ST_ ‡
Skutustasir	Sjonaripa	399,054(143,533–3,146,688)	171(171–295,820)	0.0124(0.0004–0.6404)	0.0001(0.0001–0.1585)	0.24707 (0, 0)
Spiterstulen	Skutustasir	594,637(33,123–3,152,997)	2,011(158–277,773)	0.0020(0.0004–0.5740)	0.0678(0.0009–1.2382)	0.22856 (1, 5)
Spiterstulen	Sjonaripa	610,410(58,360–3,146,688)	3,220(1,078–276,972)	0.0024(0.0002–0.2267)	0.0001(0.0001–0.1081)	0.34948 (2, 5)
Plech	Skutustasir	80,442(14,196–3,152,997)	85,266(15,970–499,974)	0.0051(0.0051–1.6749)	0.0177(0.0003–0.3581)	0.26939 (2, 4)
Plech	Sjonaripa	976,341(33,123–2,900,631)	77,142(276–496,136)	0.0016(0.0016–0.9656)	0.0002(0.0002–0.1220)	0.31694 (4, 4)
Stolberg	Spiterstulen	323,344(48,896–3,140,379)	23,370(171–307,256)	0.0099(0.0003–0.2843)	0.0070(0.0002–0.2126)	0.51614 (6, 6)
Plech	Spiterstulen	61,514(4,731–3,124,606)	65,707(276–513,144)	0.1254(0.0014–0.8335)	0.0032(0.0002–0.0679)	0.40214 (6, 6)
Plech	Stolberg	55,205(23,659–3,146–688)	75,329(29,127–498,935)	0.8361(0.0044–4.9334)	0.0978(0.0012–1.3421)	0.22454 (6, 6)
Stolberg	Haselbrunn	36,278(7,886–3,152,997)	359,332(50,828–439,866)	0.1568(0.0010–1.5267)	0.9515(0.0049–8.1789)	0.21267 (4, 6)
Stolberg	Neutras	26,814(14,196–3,152,997)	116,154(41,969–440,392)	0.0995(0.0007–0.9549)	0.1249(0.0011–1.5083)	0.2171(6, 6)
Plech	Haselbrunn	111,987(33,123–2,610,410)	90,037(19,282–490,339)	0.0498(0.0100–17.360)	0.4709(0.1205–18.385)	0.05397 (1, 5)
Plech	Neutras	67,823(33,123–3,152,997)	87,907(13,289–519,795)	0.0083(0.0083–10.080)	0.0637(0.0021–3.1174)	0.08093 (3, 6)
Haselbrunn	Neutras	2,366(263–525,500)	24,198(210–24,198)	1.6996(0.3766–3.8714)	0.0806(0.0013–2.1312)	0.11275 (2, 6)
Plech	Schaerftal	105,678(23,659–3,149,842)	79,982(25,276–556,349)	0.5737(0.0283–2.9609)	0.3098(0.0028–2.6350)	0.14292 (5, 4)
Haselbrunn	Schaerftal	36,278(14,196–3,143,533)	90,431(27,419–590,208)	0.2542(0.0014–1.9411)	0.6495(0.0023–3.2520)	0.15597 (4, 6)
Schaerftal	Neutras	2,050(11,041–3,143,533)	315(315–570,597)	0.5053(0.0701–2.1443)	0.0012(0.0012–0.6676)	0.18287 (3, 6)

* Point estimates from IMa are reported from data compiled from three separate runs. HPD-90 s are given in parentheses.

‡ Average of the estimates across all 6 loci from Arlequin.

The numbers of sites that were significant for Ф_ST_ (first) and for the exact tests of differentiation (second) are given in parentheses.

**Figure 5:**
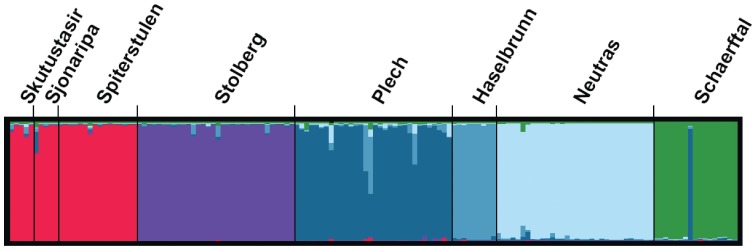
Population structure as inferred from the best fit number of groupings in STRUCTURE. Best fit model had K = 6 populations. Each color represents population assignment inferred from best fit STRUCTURE output. Individuals are shown as a separate line in the box of the population in which they were collected as labeled on top.

The best fit number of populations is six (K = 6), as estimated in STRUCTURE (Figure 5), with the probability of K = 6 of 1 and the probabilities of K = 5 and K = 7 of 10^−34^ and 10^−28^ respectively. The two Icelandic populations group with the Spiterstulen population and all other populations group separately. The grouping of the three northern populations is due to a small number of shared haplotypes. One individual from the Schaerftal population is more similar to individuals in the Plech population. This individual was checked for placement in the haplotype networks to validate that this specific individual was not mis-assigned to the Schaerftal sampling. All alleles for this individual grouped very closely with other alleles in individuals from Schaerftal and so it was kept in the dataset.

### Demographic History

**Figure 6:**
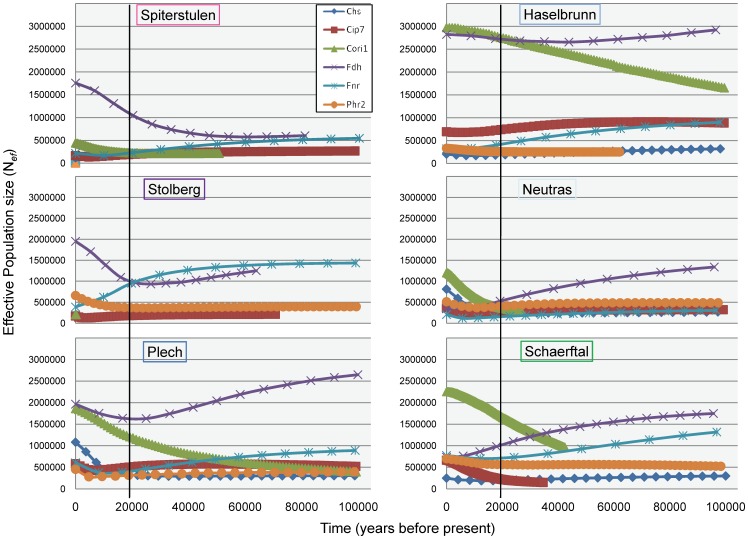
Bayesian skyline plots (BSP) showing estimates of effective population sizes (N*_ef_*) into the past for each of 6 loci. Each locus is represented by a separate line on each graph. Time (in years before the present) is on the x-axes and N*_ef_* is on the y-axes. Black lines indicate the approximate time of the LGM at 20,000 years before present.

The Bayesian skyline plots indicate expansion since the last glacial maximum (LGM, ∼20,000 years ago) in at least one locus for all populations (Figure 6). The Haselbrunn population appears to be the most stable in population size, with little change in effective population size (N*_ef_*) for all 6 loci. In contrast, the Neutras and Plech populations increased in N*_ef_* over at all 6 loci since the LGM. In all cases, expansion does not appear to be exponential, which could explain the non-significant values of Fu’s F*_S_* and Roza’s R*_2_* test statistics.

**Figure 7:**
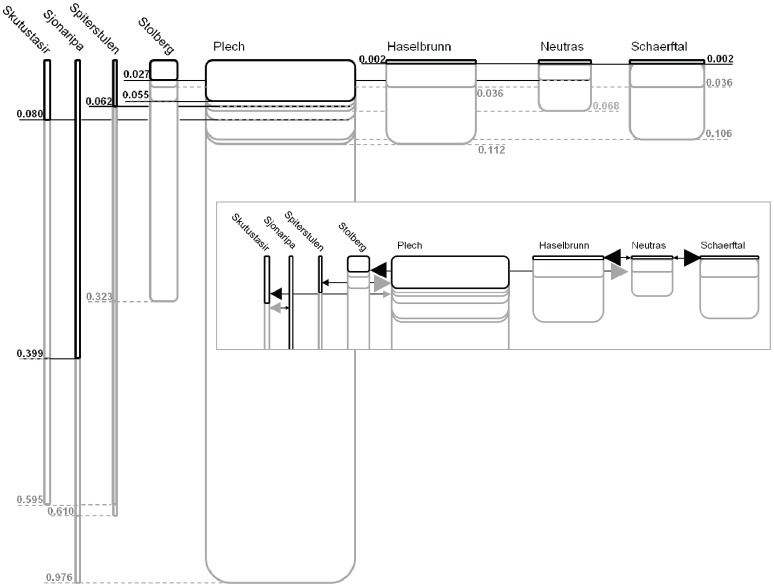
IMa results for inferred population histories. The current estimated effective population sizes are relative to the width of the box for each population. Times since divergence (in millions of years before present) are indicated by lines connecting boxes. The full black lines indicate the most recent divergence time for each population, while the dotted grey lines indicated less recent divergence times. Insert: Relative migration rates are indicated by the size of arrows. For simplicity only migration rates for the most recent divergence times for each population were included.

The best fit isolation-migration models (tested in IMa) also indicate much smaller effective population sizes (N*_ef_*) in the northern populations (Table 4 and Figure 7) versus the central populations, consistent with a recent bottleneck in the northern populations. The N*_ef_* point estimates from IMa are much lower than those estimated from the BSPs. This is most likely due to the use of the full data set for the BSP analysis and only the LNRB in IMa analysis. High rates of recombination in the data set could result in much larger N*_ef_* values when the full haplotypes are used. By using only the LNRB, we are likely underestimating N*_ef_* values in IMa.

**Table 4:** Estimates of current effective population sizes (N_ef_) inferred from the best fit model in IMa.

Population	N*_ef_*
Skutustasir	399 (13–20,202)
Sjonaripa	298 (13–20,886)
Spiterstulen	253 (13–16,364)
Stolberg	1,662 (118–18,244)
Plech	21,096 (3,247–48,699)
Haselbrunn	12,523 (2,050–48,883)
Neutras	6,733 (683–26,196)
Schaerftal	11,580 (2,524–48,751)

Point estimates are averages across all pairwise comparisons. The highest and lowest values for 90% of the highest probability densities are given in parentheses.

The divergence time point estimates included in the best fit isolation-migration models indicate deeper population divergence times for the northern populations, well before the LGM (Table 3 and Figure 7). This pattern is consistent with an earlier divergence of the northern populations from the central populations that occurred before the LGM and maintained small populations in refugia throughout the Pleistocene. All three central German populations and the Austrian population likely diverged from each other much more recently. The divergence times between the Haselbrunn, Neutras and Schaerftal populations are only approximately 2,000 years before present. Interestingly, this is in contrast with the expectation that these populations have remained stable in population size for a long period of time. It is likely that our estimates of divergence times (T) in the best fit isolation-migration model are underestimates due to the use of LNRB for this analysis. Because the rates of recombination are high in this group, very short blocks (Table S1) whose polymorphisms are representative of a more recent history than the full length sequence are used. The full sequences have additional polymorphisms that have been broken up by recombination, a signature of more time since divergence (Table S2). Therefore, it is possible that the central populations have an older divergence time than what is represented in this dataset. However, this more recent history is also reflected in the northern populations, so relative divergence should be unaffected by using the LNRB.

## Discussion

Here we present evidence of differing demographic histories amongst European populations of *A. l. petraea*. Northern European populations have lower nucleotide diversity, haplotype diversity, and recombination rates than central populations, indicative of smaller effective population sizes. Best fit isolation-migration models (IMa) and Bayesian skyline plots (BSP) of population size changes over time also indicate much lower N*_ef_* values for the northern populations. These estimates coincided with what we expect based on studies using other markers [5], [17], [18]. BSPs showed increases in effective population sizes since the LGM for all populations, including the central European populations that were cited as stable using microsatellite loci [5]. Our finding is in agreement with allozyme data [28] that indicated an excess of heterozygosity in several northern and central European populations. The difference between microsatellite markers and coding genes could be due to differences in the time scales represented by these markers. Microsatellites detect a much more recent history than do nuclear loci, which could be why expansion in the central European populations was not detected. There could also be discrepancies due to high levels of recombination which can confound the mutation model for microsatellites. The level of recombination could also account for the discrepancy of N*_ef_* estimates between IMa and BSPs. IMa uses LNRB, which have a reduced amount of polymorphism, while BSPs use the full sequence. High recombination rates are likely not restricted to the central European populations, but may not be detected in northern populations due to lower levels of nucleotide diversity. Therefore, northern populations may have expanded at a greater rate since the LGM, but detection is difficult at slow evolving loci with low levels of starting variation due to a strong bottleneck.

Haplotype networks show varying patterns of haplotype distributions among populations. By rooting networks with *A. thaliana* ecotypes, differences in the distribution of ancestral alleles among populations can be inferred. The distribution of ancestral alleles supports the theory that northern populations survived the Pleistocene as separate, possibly transitory refugia (as proposed in [28]). These refugia could be maintained as seeds in the seed bank through the Pleistocene and established new populations when the glaciers receded, although the potential for seeds to survive under ice sheets for long time spans has not been tested. Post-glacial colonization would result in all haplotype networks being anchored to *A. thaliana* through the parental population’s haplotype, with northern populations only connected through the central parent population. The patterns seen reflect older divergence times between populations with drift and mutation randomly fixing alleles.

Consistent with the patterns from the haplotype networks, the best fit isolation-migration models (IMa results) support pre-glacial divergence between northern and central populations. However, the best fit model suggests post-glacial divergence between the central European populations. These estimates contrast with those from a study that used the Plech population as a reference population in demographic modeling [17]. Our demographic model did not assume stability in any of the tested populations, allowing for a more comprehensive model.

It has been suggested that the center of origin for *A. l. petraea* is in Austria rather than Germany [28]. Haplotype networks show Schaerftal alleles as present in the most common haplotype groups and shared haplotypes with all other populations. However, recent divergence time estimates between the Schaerftal population and the central German populations in the best fit isolation-migration model makes it difficult to determine which population is the oldest. Sampling of additional Austrian populations is needed to determine if Austria is in fact the center of origin for this species.

Population connectivity revealed in STRUCTURE and the molecular covariance estimate of variance due to among population diversity (AMOVA, Ф_ST_ estimates) indicate strong population structure between populations and low levels of gene flow for all except the three northern populations. However, the best fit isolation-migration models estimate higher rates of gene flow and more recent divergence times amongst the central populations. This discrepancy amongst algorithms is likely due to high within-locus recombination rates in the central populations, resulting in an increase of private alleles in the central populations that are not detected when using the longest non-recombining block of sequence for IMa analyses. The underlying models for these algorithms are also very different in that IMa uses a coalescent approach for allele divergence while STRUCTURE uses current haplotype frequencies to determine relatedness of populations. The grouping of northern populations in STRUCTURE is likely due to a small number of shared haplotypes between these populations, however most alleles in each population are diverged. When a mutation model is incorporated into IMa, the number of mutations between the diverged haplotypes gives a more accurate estimate of divergence. Therefore, our data supports pre-glacial divergence between northern populations. This finding is consistent with high divergence rates between Scandinavian and Icelandic populations of *A. l. petraea* [5].

It is important to note that we cannot rule out possible gene flow from a third population in the pairwise population analyses. Isolation-migration model fitting in IMa assumes no gene flow from a third population. A simulation study [55] has shown that these violations can result in increases in N*_ef_* for the population that is receiving alleles from a third population and increases in the range of the highest probability density (HPD-90) intervals for N*_ef_*A and T, but with little impact on point estimates for these two parameters. Only very high values of gene flow from a third population (N*_ef_m* >0.5) have a strong impact on estimates [58]. Point estimates from the Icelandic and Norwegian populations do not show migration rates high enough to impact other comparisons, but some point estimates for the German and Austrian populations indicate that gene flow could skew values. In most pairwise comparisons, the HPD-90 intervals include values of greater than 0.5 for N*_ef_m*. The overall impact of gene flow from a third population is difficult to estimate and this violation is most likely the reason for high HPD-90 intervals for N*_ef_*A and divergence time (T) in all runs.

Gene sequences of nuclear loci complement patterns of demographic histories for European populations of *A. l. petraea* reported at microsatellite loci [5]. The differences found between marker types are important for understanding how the level of starting variation can determine the extent to which demography can impact coding genes across the genome. Including more loci (the total 23 developed in the original screen) would result in sampling a larger range of the original standing levels of genetic variation. This larger sample of loci would allow for the detection of shifts in variation seen at neutral loci due to more specific demographic factors. Including more nuclear loci would also allow for the detection of deeper histories when using only the LNRB for analysis.

Another important factor governing genome-wide genetic diversity is the level of gene flow from diverged populations or closely related species. Gene flow between *A. l. lyrata* and *A. halleri* since initial divergence has impacted the level of diversity on a genome-wide scale [59]. It would be interesting to test gene flow between *A. l. petraea* and other closely related species. Of special interest is *A. arenosa*, which maintains a stable hybrid zone with *A. l. petraea* in Austria (Koch, personal communication). It would be informative to know if there are regions of the genome undergoing different levels of gene flow and if those regions coincide with regions that are differentially shared between *A. l. lyrata* and *A. halleri*. These regions might harbor ‘speciation genes’ or genes that maintain reproductive isolating barriers. This approach would be a nice complement of candidate gene approaches for understanding mechanisms of speciation in plants (see [60] for review).

This study is one of few to describe the multi-locus impacts at coding genes under alternative demographic scenarios in the same species [6], [10], [19]. The coding genes used in this study are not completely neutral because there is selective pressure to maintain function; however they are not candidates for selection and adaptation differentiating populations. Although theory on the subject of demographic impacts on genome-wide diversity is well accepted, solid empirical data from natural populations is lacking. Understanding how genome-wide diversity is impacted by neutral processes is critical to deciphering patterns caused by non-neutral processes. Therefore, a full understanding of the demographic history of model systems, such as *Arabidopsis*, is pivotal to utilizing these systems to their full extent. The outcome of these studies will also be useful in predicting the outcomes of contemporary demographic changes in natural populations, whose impact on genetic diversity will be difficult to fully assess for many years.

## Supporting Information

Table S1 **Single Copy Nuclear Loci (SCNL) information.**
(XLSX)

Table S2 **Diversity information for full length and LNRB for each of the 6 loci.**
(XLSX)
